# Whole-Genome Sequencing of the Opportunistic Yeast Pathogen *Candida inconspicua* Uncovers Its Hybrid Origin

**DOI:** 10.3389/fgene.2019.00383

**Published:** 2019-04-25

**Authors:** Verónica Mixão, Antonio Perez Hansen, Ester Saus, Teun Boekhout, Cornelia Lass-Florl, Toni Gabaldón

**Affiliations:** ^1^Centre for Genomic Regulation, Barcelona Institute of Science and Technology, Barcelona, Spain; ^2^Department of Experimental and Health Sciences, Universitat Pompeu Fabra, Barcelona, Spain; ^3^Division of Hygiene and Medical Microbiology, Innsbruck Medical University, Innsbruck, Austria; ^4^Westerdijk Fungal Biodiversity Institute, Utrecht, Netherlands; ^5^Institute of Biodiversity and Ecosystem Dynamics, University of Amsterdam, Amsterdam, Netherlands; ^6^Institució Catalana de Recerca i Estudis Avançats, Barcelona, Spain

**Keywords:** *Candida inconspicua*, hybrid, yeast, pathogen, genome

## Abstract

Fungal infections such as those caused by *Candida* species are increasingly common complications in immunocompromised patients. The list of causative agents of candidiasis is growing and comprises a set of emerging species whose relative global incidence is rare but recurrent. This is the case of *Candida inconspicua*, which prevalence has increased 10-fold over the last years. To gain novel insights into the emergence of this opportunistic pathogen and its genetic diversity, we performed whole genome sequencing of the type strain (CBS180), and of 10 other clinical isolates. Our results revealed high levels of genetic heterozygosity structured in non-homogeneous patterns, which are indicative of a hybrid genome shaped by events of loss of heterozygosity (LOH). All analyzed strains were hybrids and could be clustered into two distinct clades. We found large variability across strains in terms of ploidy, patterns of LOH, and mitochondrial genome heterogeneity that suggest potential admixture between hybrids. Altogether, our results identify a new hybrid species with virulence potential toward humans and underscore the potential role of hybridization in the emergence of novel pathogenic lineages.

## Introduction

Fungal infections are an increasingly common problem in hospital environments, very often leading to patient’s death (Pfaller and Diekema, 2007; Lass-Flörl, 2009; Brown et al., 2012; Gabaldón and Carreté, 2016). Historically, *Candida* species have been the most common causative agents of hospital-acquired fungal infections (Pfaller and Diekema, 2007; Lass-Flörl, 2009; Brown et al., 2012; Gabaldón and Carreté, 2016). Patients at particular risk include those in the intensive care unit, those undergoing surgery, and patients with solid tumor or hematological malignancy (Lass-Flörl, 2009). Reported mortality rates for candidemia range from 28 to 59% in European surveys and depend on species, underlying disease conditions, and geographical location (Jordà-Marcos et al., 2007; Lass-Flörl, 2009). *Candida albicans* is the most common cause of candidemia, accounting for more than 50% of the cases, followed by *Candida glabrata* and *Candida parapsilosis* (Holzheimer and Dralle, 2002; Jordà-Marcos et al., 2007; Pfaller and Diekema, 2007). However, the epidemiology of candidemia has shifted in recent years, with the incidence of rare species becoming increasingly important in the clinical setting (Lass-Flörl, 2009; Pfaller et al., 2012; Sardi et al., 2013; Gabaldón and Carreté, 2016; Gabaldón et al., 2016; Bretagne et al., 2017). For instance, *Candida auris* is an emerging multi-drug resistant pathogen responsible for many outbreaks all over the world in the last few years (Forsberg et al., 2019).

*Candida inconspicua* was firstly described as *Torulopsis inconspicua* in Lodder and Kreger-van Rij (1952) and later reclassified in *Candida* (Yarrow and Meyer, 1978). The species belongs to the *Pichia cactophila* clade, together with *Pichia kudriavzevii* [synonym *Candida krusei* (Douglass et al., 2018)], *Pichia norvegensis, P. cactophila*, and *Pichia pseudocactophila* (Kurtzman et al., 2011). *C. inconspicua* is genetically similar and phenotypically identical to *P. cactophila* and it has been suggested that they represent different sexual stages of the same species (Kurtzman et al., 2008, 2011; Guitard et al., 2015). *C. inconspicua* has also been misidentified as other members of the clade such as *P. norvegensis* (Majoros et al., 2003; Guitard et al., 2015). Many studies support that *C. inconspicua* can often be found in lactic products, including milk, cheese, or butter (Minervini et al., 2001; Suzzi et al., 2003; Krukowski et al., 2006; Callon et al., 2007; Kurtzman et al., 2011). In addition, it has recently been reported in traditional alcoholic beverages such as oil palm wine and a sorghum beer called *tchapalo* (Egue et al., 2018).

*Candida inconspicua* is also responsible for clinical infections, more prominently in European countries (Majoros et al., 2005; Pfaller et al., 2010; Guitard et al., 2013). A more than 10-fold increase in *C. inconspicua* infections between 1997–2000 and 2001–2004 (increase of 9 to 276 cases) followed by an apparent stabilization has been reported by a multi-center study (Pfaller et al., 2010). The majority of *C. inconspicua* infections are associated with osteomyelitis, oropharyngeal and esophageal candidiasis in HIV positive patients, as well as with candidemia in patients with hematological malignancies (Majoros et al., 2005). Frequently, *C. inconspicua* isolates derive from colonization of the digestive and respiratory tracts from unknown sources (Guitard et al., 2013). However, the above mentioned reported isolations make contaminated milk or other food products a possible source for the infecting strains. *C. inconspicua* was previously described as presenting a low susceptibility to fluconazole and other antifungal agents (Majoros et al., 2005; Pfaller et al., 2010; Guitard et al., 2013). For instance, Pfaller et al. (2010) reported that, depending on the site of isolation, the frequency of fluconazole resistant strains could range between 26.1% (skin and soft tissue) and 62.9% (genital tract), thus indicating a high phenotypic heterogeneity among *C. inconspicua* isolates.

To shed light on the genetic makeup and diversity of this emerging opportunistic pathogen we undertook the whole genome sequencing and assembly of the type strain and compared the genomic sequences of 10 other clinical isolates. We found that *C. inconspicua* has a highly heterozygous genome with patterns suggestive of a hybrid origin. We discuss this finding in comparison with two other medically important *Candida* hybrid lineages: *Candida metapsilosis* and *Candida orthopsilosis* (Pryszcz et al., 2014, 2015; Schröder et al., 2016). Following *C. metapsilosis*, *C. inconspicua* is the second reported case of an opportunistic *Candida* human pathogen for which all clinical strains analyzed so far are hybrids, suggesting that hybridization may be at the root of its ability to infect humans (Mixão and Gabaldón, 2018).

## Materials and Methods

### Library Preparation and Genomic DNA Sequencing

For this project we sequenced *C. inconspicua* type strain (CBS180), and 10 other clinical isolates. These isolates were sent by collaborating laboratories in the frame of an antifungal susceptibility test project. They were grown on Sabouraud’s 2% dextrose agar at 30°C for 72 h and re-identified using direct extraction method by MALDI-TOF (MALDI-Biotyper, Bruker, Daltonics, Database version, United States). Genomic DNA extraction of the 11 *C. inconspicua* strains was performed using the MasterPure Yeast DNA Purification Kit (Epicentre, United States) following manufacturer’s instructions. Briefly, *C. inconspicua* cultures were grown in an orbital shaker overnight (200 rpm, 30°C) in 15 ml of YPD medium. Cells were harvested using 4.5 ml of each culture by centrifugation at maximum speed for 2 min, and then they were lysed at 65°C for 15 min with 300 μl of yeast cell lysis solution (containing 1 μl of RNAse A). After being on ice for 5 min, 150 μl of MPC protein precipitation reagent were added into the samples, and they were centrifuged at 16.000 *g* for 10 min to pellet the cellular debris. The supernatant was transferred to a new tube, DNA was precipitated using 100% cold ethanol and centrifuging the samples at 16.000 *g*, 30 min, 4°C. The pellet was washed twice with 70% cold ethanol and, once the pellet was dried, the sample was resuspended in 100 μl of TE. All gDNA samples were cleaned to remove the remaining RNA using the Genomic DNA Clean & Concentrator kit (Epicentre) according to manufacturer’s instructions. Total DNA integrity and quantity of the samples were assessed by means of agarose gel, NanoDrop 1000 Spectrophotometer (Thermo Fisher Scientific, United States) and Qubit dsDNA BR assay kit (Thermo Fisher Scientific).

Whole-genome sequencing was performed at the Genomics Unit from Centre for Genomic Regulation (CRG) with a HiSeq2500 machine. Libraries were prepared using the NEBNext Ultra DNA Library Prep kit for Illumina (New England BioLabs, United States) according to manufacturer’s instructions. All reagents subsequently mentioned are from the NEBNext Ultra DNA Library Prep kit for Illumina if not specified otherwise. 1 μg of gDNA was fragmented by nebulization in Covaris to a size of ∼600 bp. After shearing, the ends of the DNA fragments were blunted with the End Prep Enzyme Mix, and then NEBNext Adaptors for Illumina were ligated using the Blunt/TA Ligase Master Mix. The adaptor-ligated DNA was cleaned-up using the MinElute PCR Purification kit (Qiagen, Germany) and a further size selection step was performed using an agarose gel. Size-selected DNA was then purified using the QIAgen Gel Extraction Kit with MinElute columns (Qiagen) and library amplification was performed by PCR with the NEBNext Q5 Hot Start 2X PCR Master Mix and index primers (12–15 cycles). A further purification step were done using AMPure XP Beads (Agentcourt, United States). Final libraries were analyzed using Agilent DNA 1000 chip (Agilent) to estimate the quantity and check size distribution, and they were then quantified by qPCR using the KAPA Library Quantification Kit (KapaBiosystems, United States) prior to amplification with Illumina’s cBot. Libraries were loaded and sequenced 2 × 125 on Illumina’s HiSeq 2500. Base calling was performed using Illumina pipeline software. In multiplexed libraries, we used 6 bp internal indexes (5′ indexed sequences). De-convolution was performed using the CASAVA software (Illumina, United States). Sequence data of the genomes has been deposited in short read archive (SRA) under the BioProject Accession No. PRJNA517794.

### *De novo* Genome Assembly and Read Mapping

Raw sequencing data was inspected with FastQC v0.11.5^1^. Paired-end reads were filtered for quality below 10 or size below 31 bp and for the presence of adapters with Trimmomatic v0.36 (Bolger et al., 2014). The K-mer Analysis Toolkit (KAT; Mapleson et al., 2017) was used to get the GC content and *k*-mer frequency of CBS180 reads and estimate the expected genome size. SPAdes v3.9 (Bankevich et al., 2012) was used to perform the genome assembly using this strain. Afterward, redundant contigs were removed with Redundans v0.13c (Pryszcz and Gabaldón, 2016) using default parameters, i.e., 51% minimum identity and at least 80% overlap. The quality of the assembly was inspected with Quast v4.5 (Gurevich et al., 2013) and KAT (Mapleson et al., 2017). Genome annotation was performed with Augustus v3.1 (Stanke and Morgenstern, 2005), using *C. albicans* as model organism. BUSCO v3 (Waterhouse et al., 2017) was used to assess the completeness predicted proteome considering the Ascomycota database. This genome assembly and annotation have been deposited at DDBJ/ENA/GenBank under the Accession No. SELW00000000. The version described in this paper is version SELW01000000.

Phylome reconstruction was performed using the PhylomeDB pipeline (Huerta-Cepas et al., 2014) as described by Pryszcz et al. (2015), using the predicted proteome as seed, and considering other twenty-one Saccharomycotina species (Table A in Supplementary file 1). A second phylome considering only proteins predicted in scaffolds > 10 kb was also reconstructed to confirm the obtained results. The presented results correspond to the phylome considering all proteins. *C. inconspicua* phylomes are available in PhylomeDB (Huerta-Cepas et al., 2014) with the ID 454 and 498 (this one only considering scaffolds > 10 kb). Gene gain and loss analysis in seed branch was performed based on the phylome results. A BLASTp (Zhang et al., 2000) was performed against the UniProt database, in order to determine the possible function associated to these genes, as well as their GO terms. An enrichment analysis was done using FatiGO (Al-Shahrour et al., 2004), and the results were summarized with REVIGO (Supek et al., 2011).

Read mapping for all strains (Table 1) was performed with BWA-MEM v0.7.15 (Li, 2013). Picard v2.1.1^2^ was used to sort the resulting file by coordinate, as well as to mark duplicates, create the index file, and obtain mapping statistics. Mapping results were inspected with IGV version 2.0.30 (Thorvaldsdóttir et al., 2013). Mapping coverage was determined with SAMtools v0.1.18 (Li et al., 2009).

**Table 1:** List of *Candida inconspicua* strains used in this project, with indication of their respective clade, place of collection, specimen, number of heterozygous variants, level of loss of heterozygosity, and estimated overall ploidy.

Strain	Clade	Country	Specimen	Heterozygous	Estimated	nQuire most
				variants/kb	LOH	probable
					(>100 bp)	ploidy
14ANR23920	Clade 1	Germany	Blood	14.66	65.18%	Diploid
9_16	Clade 1	Belgium	Blood	14.57	65.43%	Diploid
CI1	Clade 1	Austria	Abdominal fluid	14.15	66.45%	Diploid
CBS180^∗^	Clade 2	Netherlands	Sputum	14.34	65.11%	Diploid
110_10	Clade 2	Austria	Blood	18.18	55.98%	Triploid
1282	Clade 2	Romania	Swab	18.88	54.06%	Triploid
CNM_CL6867	Clade 2	Spain	Swab	19.35	53.01%	Triploid
IUM_96-0030	Clade 2	Italy	Swab	18.84	54.21%	Triploid
LL867	Clade 2	Spain	Blood	19.76	51.94%	Triploid
NRZ_BK_345	Clade 2	Germany	Blood	19.70	52.78%	Triploid
UCSC_1590	Clade 2	Italy	Blood	19.30	53.06%	Triploid


*Non-type strains were collected in the last 10 years in the framework of antifungal susceptibility testing. ^∗^Type strain.*

### Variant Calling and Ploidy Estimation

Samtools v0.1.18 (Li et al., 2009) and Picard v2.1.1^2^ were used, respectively, to index the reference and create a dictionary to be used in subsequent variant calling steps. GATK v3.6 (McKenna et al., 2010) was used to call variants with the tool HaplotypeCaller set with –genotyping_mode DISCOVERY -stand_emit_conf 10 -stand_call_conf 30 -ploidy 2 -nct 8. The tool VariantFiltration of the same program was used to filter the vcf files with the following parameters: –clusterSize 5 –clusterWindowSize 20 –genotypeFilterName “heterozygous” –genotypeFilterExpression “isHet = =1” –filterName “bad_quality” -filter “QD < 2.0 || MQ < 40 || FS > 60.0 || HaplotypeScore > 13.0 || MQRankSum < -12.5 || ReadPosRankSum < -8.0” –filterExpression “DP < = 20” –filterName “DepthofQuality.” In order to determine the number of SNPs/kb, a file containing only SNPs was generated with the SelectVariants tool. Moreover, for this calculation only positions in the reference with 20 or more reads were considered for the genome size, and these were determined with bedtools genomecov v2.25.0 (Quinlan and Hall, 2010).

To estimate the ploidy of each strain, nQuire histotest (Weiß et al., 2018) was used to test which distribution (diploid, triploid, or tetraploid) fits better to the variant frequency data. Given that for some of the strains the results were not clear, the allele frequency of each heterozygous variant was calculated by dividing the number of reads supporting the alternative haplotype by the total of reads mapping at that position. Allele frequency density was plot for each strain considering only scaffolds with more than 100 kb.

### Determination of the Different Hybridization Events and Parental Divergence

To determine heterozygous and loss of heterozygosity (LOH) blocks, the procedure applied and validated by Pryszcz et al. (2015) was used. Briefly, bedtools merge v2.25.0 (Quinlan and Hall, 2010) with a window of 100 bp was used to define heterozygous regions, and by opposite, LOH blocks would be all non-heterozygous regions in the genome. The minimum LOH and heterozygous block size was established at 100 bp. Due to the aneuploidies observed for *C. inconspicua*, contrary to what Pryszcz and colleagues performed, no coverage filter was applied.

As mentioned in section “Results,” all *C. inconspicua* strains analyzed here were found to be hybrids. The current divergence between the parental genomes was calculated dividing the number of heterozygous positions by the total size of heterozygous blocks. Another important step was to check whether all strains were originated from the same hybridization event. For this, pairwise comparisons between overlapping LOH blocks were performed using bedtools jaccard v2.25.0 (Quinlan and Hall, 2010), which allowed us to get the number of nucleotides in the intersection of the two strains over the number of nucleotides present in their union. Moreover, assuming that LOH blocks with exactly the same boundaries are not independent events, it was decided to repeat the pairwise comparisons, but this time instead of analyzing the number of nucleotides in LOH regions in both strains, it was decided to get the number of LOH blocks with exactly the same boundaries in both strains. In this case, in order to avoid false positives, short LOH blocks (<1 kb), as well as very short scaffolds (<10 kb) were not considered for the analysis.

### Phylogenetic Analysis

To obtain the phylogenetic relationship between the 11 strains, FastaAlternateReferenceMaker tool of GATK v3.6 (McKenna et al., 2010) was used to obtain the genome sequence of each strain substituting each position with a homozygous SNP by the respective allele. Furthermore, bedtools subtract and bedtools getfasta v2.25.0 (Quinlan and Hall, 2010) were used to remove from these new sequences all positions with a heterozygous SNP or an INDEL in at least one of the strains. In the end, as INDELs were not considered for the analysis, the sequences of the 11 strains presented exactly the same size, constituting a sequence alignment of 9,971,439 bp. A Maximum-likelihood tree representative of this alignment was obtained with RAxML v8.2.8 software (Stamatakis, 2014), using the GTRCAT model. The same approach was applied to obtain a phylogeny for MAT locus.

### Lineage Prediction and Detection of Recombination

To predict the number of lineages and clusters in our dataset, as well as to detect traces of admixture between the different strains, we used fastGEAR (Mostowy et al., 2017). For that, this program was set to complete mode, and the same alignment used for the phylogenetic analysis was given as input. All the other parameters were set to default. To have a control of the two expected scenarios, we decided to do the same analysis for *C. metapsilosis* and *C. orthopsilosis*, as representatives of a unique hybridization event and multiple hybridization events, respectively (Pryszcz et al., 2015; Schröder et al., 2016). In both situations, all Illumina paired-end sequencing libraries available (BioProjects PRJEB4430, PRJEB1698 and PRJNA322245), as well as five extra libraries for *C. metapsilosis* and two other libraries for *C. orthopsilosis*, which will be soon publicly available under the BioProject PRJNA520893 (manuscript in preparation) were used for read mapping, post-processing analysis, variant calling and sequence alignment as mentioned before for *C. inconspicua*.

### Mitochondrial Genome Assembly

The mitochondrial genome assembly for *C. inconspicua* was performed using the filtered Illumina paired-end reads of CI1 strain. NOVOPlasty v2.7.2 (Dierckxsens et al., 2017) with default parameters was used to assemble this genome, taking as seed input *C. inconspicua Cox2* gene (Accession No. EF599394.1). A final 31 kb contig was obtained. NUCmer algorithm of MUMmer v3.1 (Kurtz et al., 2004) was used to align this final assembly against *Pichia kluyveri* mitochondrial genome (Accession No. NC_022158.1). MUMmerplot algorithm of MUMmer v3.1 (Kurtz et al., 2004) was used to visualize this alignment and see that our final 31 kb scaffold covers a big part of *P. kluyveri* mitochondrial genome (Supplementary Figure 1). Mitochondrial genome annotation was performed with MITOS2 (Bernt et al., 2013). Read mapping and variant calling of all strains against this final mitochondrial assembly was performed as mentioned before. The mitochondrial genome of each strain was reconstructed with FastaAlternateReferenceMaker tool of GATK v3.6 (McKenna et al., 2010), using IUPAC code to solve heterozygous positions. A NJ phylogenetic tree was generated with SplitsTree v4 (Huson and Bryant, 2006). To compare the topology of mitochondrial and nuclear trees, RAxML v8.2.8 software (Stamatakis, 2014) was used to compute per site log Likelihoods for each tree given each of the alignments. Consel v1.2 (Shimodaira and Hasegawa, 2001) was used to assess the confidence that a given tree could correspond to a given alignment.

### Antifungal Susceptibility Test

To test whether the different clades of *C. inconspicua* presented different susceptibilities to antifungal drugs, we performed antifungal susceptibility tests on all 11 strains using two different methods (Etest gradient strips and EUCAST). By EUCAST broth-microdilution, which is one of the main international reference methods, the following drugs were tested: Itraconazole (Sigma, Rowville, Australia), Posaconazole (Schering-Plough, Kenilworth, NJ, United States), Isavuconazole (Basilea, Basel, Switzerland), Fluconazole (Sigma), Voriconazole (Sigma), Anidulafungin (Pfizer, New York, NY, United States), Micafungin (Astellas, Munich, Germany), Caspofungin (Sigma), and Amphotericin B (Sigma) in Cellstar plates (Cellstar Cat-No. 655180, Greiner Bio-One, United States). Pre-cultures were grown on Sabouraud’s 2% dextrose agar at 30°C for 24 h for all method used. The RPMI used for the different media was provided by Sigma (RPMI-1640 Medium, R6504-50L). Broth-microdilution was performed according to EUCAST guidelines (Arendrup et al., 2017) with minor modifications. To ensure proper growth, the incubation time was prolonged to 48 h, and the optical density threshold of the plate reader reading was lowered to 0.1. Plates were evaluated at 48 h both visually and by plate reader (Microplate Reader model 680, Bio-Rad, United States). *Candida parapsilosis* ATCC 22019 or *P. kudriavzevii* ATCC 6258 were used as quality control.

On the other hand, a commercial test was also used. Specifically, Etest strips with Itraconazole, Posaconazole, Isavuconazole, Fluconazole, Voriconazole, Anidulafungin, Micafungin, Caspofungin, and Amphotericin B (all bioMérieux SA, France, except Isavuconazole, which was provided by Liofilchem, Italy) were used as indicated by the respective manufacturers. Plates were incubated at 37°C and visually read after 48 h to coincide with the EUCAST conditions.

## Results

### Evidence for the Hybrid Nature of *C. inconspicua*

**FIGURE 1:**
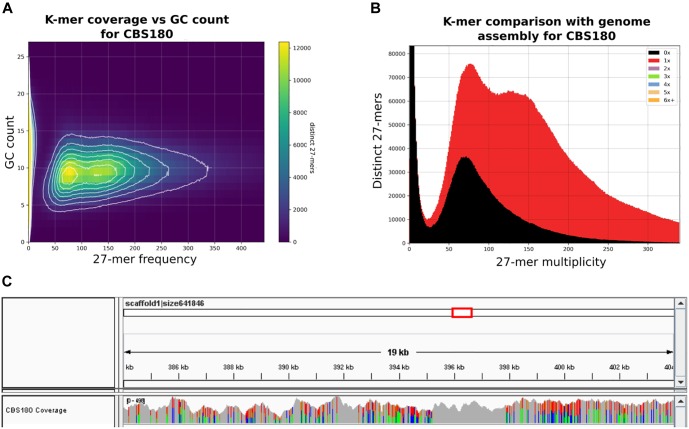
Heterozygosity patterns in *Candida inconspicua* type strain genome. **(A)** 27-mer frequency in CBS180 genomic reads and respective GC content. **(B)** 27-mer frequency in CBS180 genomic reads and indication of their presence (red) or absence (black) in the genome assembly. Although both 27-mer count peaks were represented in the genome assembly, roughly half of the reads corresponding to the first one was excluded. These reads probably correspond to redundant heterozygous contigs that were intentionally removed during the reduction step of the assembly process (see section “Materials and Methods”). **(C)** IGV coverage track of a 19 kb region of *C. inconspicua* scaffold 1. Colors represent polymorphic positions. Highly heterozygous regions are clearly interspaced by blocks of loss of heterozygosity (LOH).

To uncover the genomic features of *C. inconspicua* we sequenced the type strain CBS180 using an Illumina-based, pair-end sequencing strategy (see section “Materials and Methods”). GC content and 27-mer count analyses of the sequencing reads revealed two peaks with similar GC content but different coverage (Figure 1A,B). The first peak presented roughly half of the coverage of the second (Figure 1B), and therefore it could correspond to highly heterozygous regions of a diploid genome. We next performed a *de novo* genome assembly using SPAdes (Bankevich et al., 2012) and redundans (Pryszcz and Gabaldón, 2016), an assembly pipeline tailored for highly heterozygous genomes (see section “Materials and Methods”). The final assembly comprised 10,353,411 bp divided in 744 contigs (76 longer than 50 kb, representing 80.16% of the genome) with 35.1% GC content and a N50 of 100,257 bp. Genome annotation predicted 5,079 proteins (see section “Materials and Methods”). The final genome size and the number of proteins that we have obtained are similar to what was previously described for the closely related species *P. kudriavzevii* (10.9 Mb and 4,949 predicted proteins; Cuomo et al., 2017), suggesting the completeness of the *C. inconspicua* genome. Indeed, 99.13% of CBS180 reads aligned to the assembly, and the missing 27-mers were in heterozygous regions, possibly corresponding to redundant contigs (Figure 1B). Furthermore, our predicted proteome has a 89% completeness as assessed by BUSCO (see section “Materials and Methods”). Phylome reconstruction (Gabaldón, 2008) in the context of twenty-one other Saccharomycotina species (see section “Materials and Methods” and Table A in Supplementary File 1) identified 501 species-specific genes. Furthermore, genes specifically duplicated in *C. inconspicua* seemed to be enriched in transmembrane transport and drug export functions, among others (Table B in Supplementary File 1).

Mapping of CBS180 reads against its own genome assembly followed by variant calling showed the presence of 14.36 variants/kb, of which most (14.34 variants/kb) corresponded to heterozygous positions (Table 1 and Supplementary File 2). Importantly, these variants were not homogeneously distributed throughout the genome, but rather formed stretches of highly heterozygous sequences separated by what appeared to be LOH, as it has been observed in previously analyzed genomes from hybrids of the *Candida* clade (Pryszcz et al., 2014, 2015; Schröder et al., 2016). These LOH blocks were flanked by heterozygous blocks with relatively constant levels of heterozygosity (36.4 heterozygous variants/kb). An illustrative example of such patterns is presented in Figure 1C. The high level of sequence divergence between the two haplotypes in the heterozygous blocks (3.64%) is much higher than the divergence observed between most distantly related strains of well-recognized yeast species (i.e., 1.1% for *Saccharomyces cerevisiae*) (Peter et al., 2018). Altogether, these analyses highly suggested that *C. inconspicua* type strain is a hybrid with a chimeric genome.

### Genome Heterogeneity Across *C. inconspicua* Strains

To gain a better insight into this species, 10 other clinical strains were sequenced, and their sequencing reads were mapped against the reference genome assembly described above (Table 1). All analyzed strains were highly heterozygous, with 14.15–19.76 heterozygous variants/kb, and all of them presented highly heterozygous genomic regions interspersed with LOH blocks (Table 1, Supplementary File 2 and Supplementary Figure 2). Thus, similar to the previously described hybrids in *C. metapsilosis* and *C. orthopsilosis* (Pryszcz et al., 2014, 2015; Schröder et al., 2016), *C. inconspicua* clinical isolates seemed to comprise a majority of hybrid strains, with all 11 strains analyzed so far being hybrids. The presence of both a and alpha mating-types in the MAT locus suggests matting as a possible origin of the hybrids (Supplementary Figure 3).

**FIGURE 2:**
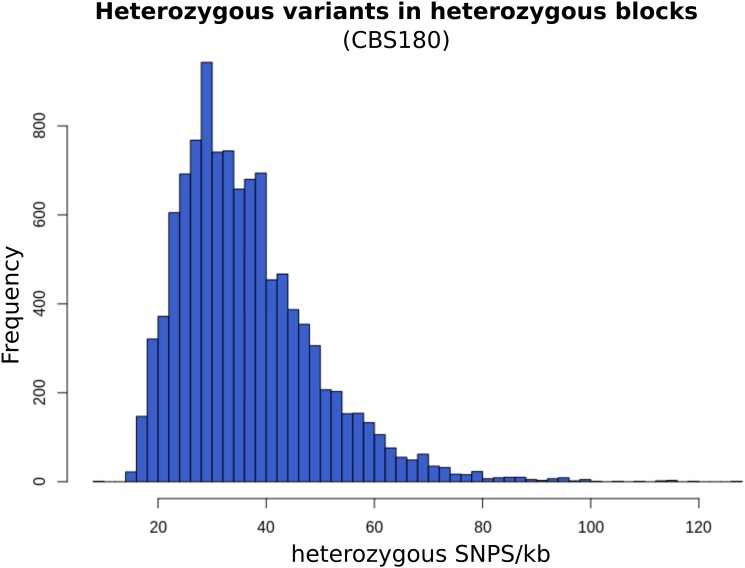
Frequency of heterozygous variants per kilo-base in CBS180 heterozygous blocks. The distribution of this frequency is close to normal, with a peak at 30 variants/kb, consistently with the estimated current parental divergence.

Similar to previous studies (Pryszcz et al., 2014), the non-homogeneous distribution of heterozygous variants throughout the genome allowed us to define blocks of heterozygosity (see section “Materials and Methods”), which correspond to regions that retain genetic material from both hybridized lineages. On average, each strain presented 12,044 heterozygous blocks with an average size of 326 bp each, overall covering 38.15% of the genome, and comprising 82.41% of the heterozygous variants (Supplementary File 2). Based on the density of heterozygous variants within heterozygous blocks, we estimated that the current sequence divergence at the nucleotide level between the two parental lineages is approximately 3.72% (Figure 2).

**FIGURE 3:**
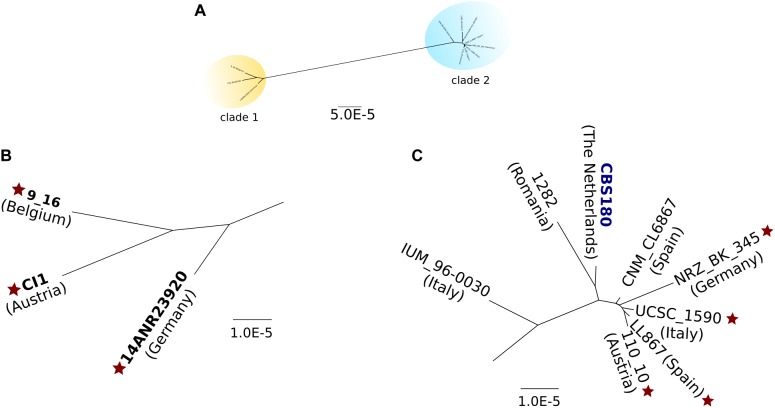
Maximum likelihood phylogeny of all *C. inconspicua* strains. A concatenated alignment of 9,971,439 bp was used to reconstruct this phylogeny. Positions with heterozygous variants or INDELs in at least one strain were removed from the analysis. The type strain is marked in dark blue. Diploid strains are highlighted in bold. Star indicates invasive strains. **(A)** Overall view of the nuclear genome phylogeny of *C. inconspicua*, with two distinct clades highlighted in yellow (clade 1) and blue (clade 2). **(B)** Closer view on *C. inconspicua* clade 1. **(C)** Closer view on *C. inconspicua* clade 2.

We next used called SNPs to reconstruct phylogenetic relationships between the sequenced *C. inconspicua* strains using a maximum likelihood approach (see section “Materials and Methods”). The resulting strain phylogeny revealed the presence of at least two distinct clades (Figure 3), with the strains 14ANR23920, CI1, and 9_16 forming one clade (clade 1) separated by a long branch from another clade comprising the remaining strains (clade 2). Within clade 2, IUM_96-0030 appeared at a relatively long distance from the rest of the clade, as did, to a lesser extent, CBS180 and 1282 (Figure 3). The two clades were not related with the geographical distribution of the different strains, but invasive strains seemed to form two clusters, one in each of the clades (Figure 3 and Table 1). Furthermore, the levels of susceptibility of each strain to azoles, echinocandins, or amphotericin B (see section “Materials and Methods”) seemed to be unrelated with their position in the phylogeny (Supplementary File 3).

The existence of two separate hybrid clades pointed to two possible scenarios: (i) a unique hybridization event followed by ancestral divergence of the two clades; or (ii) two independent hybridization events between the same parental lineages, each originating one of the clades. To explore these scenarios, we first analyzed the MAT locus of the different strains (Supplementary Figure 3). While for mating-type alpha we could identify two major clades, which coincide with the nuclear genome phylogeny, for mating-type a, a third clade formed by CBS180 and IUM_96-0030 was detected (Supplementary Figure 3). Thus, this analysis did not allow us to clearly support one or the other scenario. In a second attempt to explore these scenarios, we used a previously described approach based on the comparison of patterns of LOH blocks between strains (Pryszcz et al., 2015), where the presence of large LOH blocks with similar boundaries is indicative of a common origin. To do so, we defined LOH blocks for each *C. inconspicua* strain (see section “Materials and Methods”). On average, each strain presented 20,620 blocks with an average size of 292 bp, which covered 57.93% of the genome. Expectedly, pairwise comparisons of the overlap between LOH of each two strains revealed the same two clades as the phylogenetic analysis, but with CBS180, 1282 and IUM_96-0030 not being clearly classified to any of them (Supplementary Figure 4). To investigate whether the two clades resulted from two different hybridization events we identified LOH blocks with exactly the same boundaries in each pairwise comparison of the strains. Considering only blocks at least 1 kb-long present in scaffolds longer than 10 kb resulted in the same two clades (Supplementary File 2). However, all pairwise comparisons shared at least five large LOH blocks with exact boundaries. Indeed, disregarding any size limit in LOH blocks or scaffold size, all strains shared 633 blocks (3.07% of the average number of blocks, Supplementary Figure 5 as example). The relative low number of shared blocks did not allow us to clearly discard the possibility that more than one hybridization event have occurred. In fact, cluster and lineage prediction (see section “Materials and Methods”), using *C. metapsilosis* and *C. orthopsilosis* as control scenarios for one and more than one hybridization events, respectively, clearly identified two different lineages in *C. inconspicua* (Supplementary Figure 6). Therefore, we considered that the most probable scenario is that all these strains were originated by two hybridization events, each one generating one of the clades.

### Aneuploidies and Mitochondrial Genome Heterogeneity Suggest Possible Admixture Among *C. inconspicua* Hybrids

We next estimated the ploidy level of each *C. inconspicua* strain using patterns of allele frequency (see section “Materials and Methods”). Our results pointed to the existence of two different groups of strains. Firstly, CBS180, and the three strains of clade 1, 14ANR23920, 9_16, and CI1, were mainly diploid (*r*^2^ > 0.9, Table 1). In contrast, the remaining seven strains presented a triploid model as the one with best support, although the results were inconclusive (Supplementary File 4). A closer inspection of allele frequency plots for individual scaffolds suggested the existence of a large number of aneuploidies, as shown by the coexistence of patterns compatible with tetraploid, triploid and diploid configurations, or a mixture thereof, in different scaffolds of a given strain (Supplementary Figure 7). The number of such aneuploidies was very reduced in the first group of strains with a largely diploid structure. All strains with large levels of aneuploidies belonged to the phylogenetic clade 2. In this regard, the type strain (CBS180) was the only diploid strain within a clade consisting mostly of aneuploid strains. This anomaly was perhaps related to the fact that the type strain has been conserved in isolated culture for a long time, likely promoting a fast diploidization, due to its frequent subculturing. Aneuploidies in the other strains may indicate intermediate levels of diploidization from an allotetraploid parental.

In another attempt to clarify the origin of these hybrid strains, we assembled a 31 kb region of *C. inconspicua* mitochondrial genome (see section “Materials and Methods”). This region was obtained from CI1 strain (clade 1), as with the type strain (CBS180) we could only get a highly fragmented mitochondrial assembly (see section “Materials and Methods”). The reads of all strains were mapped against this region. Overall, the patterns of variation in the mitochondrial genome were consistent with the nuclear genome phylogeny (Figure 3 and Supplementary Figures 8, 9), with most polymorphisms likely resulting from accumulation of SNPs through time in the same mitochondrial genome background, rather than representing two different mitochondrial genomes each coming from a different parental species of the hybrids. Interestingly, mitochondrial genomes from all strains of clade 2 revealed some short deleted regions, from which we highlight a major 1.5 kb deletion in *Cox1* (Supplementary Figure 8), corresponding to a LAGLIDADG endonuclease domain, which is a mobile element.

As expected for a scenario of hybridization, the phylogenetic tree of the mitochondrial genome (Supplementary Figure 9) did not present the same topology as the one of the nuclear genome (AU test p-val < 0.033), Even so, we could identify exactly the same clades and sub-clades in the two trees a (Supplementary Figure 9). Importantly, the IUM_96-0030 strain showed heterogeneous variation patterns consistent with the presence of mitochondrial sequences from the two clades (Supplementary Figure 8). For instance, besides the presence of heterozygous SNPs in the mitochondrial sequence, this strain presented some coverage in the above mentioned 1.5 kb deletion, indicating that this strain presented heteroplasmy (i.e., presence of two mitochondrial sequences within the same cell). This suggested that this clade 2 strain may have fused with a *C. inconspicua* strain belonging to clade 1. Indeed, fastGEAR (Mostowy et al., 2017) identified recent recombination in some nuclear regions of IUM_96-0030 whose source was clade 1 (Supplementary File 5). The unbalanced representation of the two types of mitochondria in IUM_96-0030 could be related to an unbalance in the inheritance of the mitochondrial genome, which was previously postulated to occur in yeast hybrids (Verspohl et al., 2018). This pointed to the occurrence of recent admixture between different *C. inconspicua* strains. Considering this, a plausible scenario is that the aneuploidies mentioned above and the mitochondrial genome heterogeneity described here are not unrelated phenomena, and both indicate that several strains of clade 2 result from recent fusions between *C. inconspicua* hybrids.

## Discussion

*Candida inconspicua* is an opportunistic pathogenic yeast which is increasingly reported as a source of infection and often presents antifungal resistance (Sugita et al., 2004; Cendejas-Bueno et al., 2010; Guitard et al., 2013; Arendrup and Patterson, 2017). In this work, we have *de novo* assembled its type strain (CBS180) and sequenced 10 additional clinical isolates to obtain a better understanding of its recent evolution. Our results show compelling evidence for a hybrid nature of the *C. inconspicua* lineage, with all strains analyzed so far being hybrids between the same two parental lineages. Sequenced strains clearly clustered into two distinct clades. Although we consider that our results are not sufficiently conclusive, the low frequency of shared LOH blocks, and the identification of two different lineages by fastGEAR (Mostowy et al., 2017) make two independent hybridization events as the most plausible scenario.

All strains analyzed in this work were collected in Europe and the two clades do not show a particular enrichment in any geographical area. Although the inclusion of a larger number of strains may reveal geographical patterns in the future, this pattern is reminiscent of those of *C. metapsilosis*, where only a single hybrid lineage was found to have a global distribution, and *C. orthopsilosis*, where four different clades representing independent hybridization events have been identified but each of which has a widespread distribution (Pryszcz et al., 2015; Schröder et al., 2016).

Hybrid genomes present high levels of heterozygosity which may result in negative epistatic interactions and, consequently, reduced fitness (Mixão and Gabaldón, 2018). Such negative effects of hybridization can sometimes be compensated by emerging phenotypic properties that enable adaptation to a new niche, and therefore may offer a competitive advantage in certain circumstances. In any case, hybridization is generally followed by different processes that lead to a gradual stabilization of these genomes, like whole genome duplication, LOH, or partial or complete chromosome loss (Mixão and Gabaldón, 2018). In *C. inconspicua*, we could observe different aneuploidies, which might represent intermediate ploidy stages to achieve the so-called genome stabilization. For instance, we could identify two groups of strains. A first group, mainly diploid, with less heterozygous variants and a higher level of LOH, and a second one with different ploidy levels, and less LOH. These two groups are almost completely coincident with the two phylogenetic clades, except for the mainly diploid type strain CBS180, which is intermingled in a clade of mostly aneuploid strains (clade 2, Figure 3). This clear diploid status of CBS180 is atypical of strains in clade 2. Furthermore, contrary to what is generally expected for hybrid genomes, where the improper chromosome pairing can cause problems during meiosis (Mixão and Gabaldón, 2018), CBS180 is able to enter meiosis and form ascospores (Guitard et al., 2015). This might be related to the apparent genomic stabilization that we have observed for this strain. Indeed, this strain is the type strain of *C. inconspicua* and therefore is being kept in collections for many years, completely isolated from all the other strains. We cannot be certain of whether this strain was diploid when it was collected or not, but we believe that the isolated environment and the recurrent subculturing and consequent bottleneck to which this strain is subject can have helped the genome stabilization and contributed to its different genomic patterns when compared to all the other strains.

The high prevalence of aneuploidies in some *C. inconspicua* strains is unexpected when compared to the evolutionary patterns observed in other hybrids (Pryszcz et al., 2014, 2015; Schröder et al., 2016). Additionally, the analysis of their mitochondrial genomes suggests the occurrence of crosses between different *C. inconspicua* strains and enabled us to distinguish the same three sub-clades of strains within clade 2 as the nuclear phylogeny (Figure 3 and Supplementary Figure 8). For instance, a sub-clade formed exclusively by IUM_96-0030, another one formed by CBS180 and 1282, and a third one with the remaining strains of clade 2. This suggests that clade 2 is formed by three sub-clades of hybrid strains, which are now in a process of diploidization after probably independent crossing events of two *C. inconspicua* strains. A possible scenario is that, in a process similar to the *C. albicans* parasexual cycle (Forche et al., 2008), two diploid hybrid strains form a tetraploid intermediate that would subsequently lose chromosomes until a diploid state is regained. Another possible explanation is that possibly originated ascospores can eventually cross with other *C. inconspicua* strains, working as a source of variability.

## Conclusion

*Candida inconspicua* is a lineage comprising opportunistic pathogens with a hybrid origin. Although the number of tested strains is low, the absence of homozygous parentals among clinical isolates suggests that the parental lineages are/might be less able to cause infections when compared to hybrid strains. This adds to a growing list of hybrid yeast opportunist lineages and underscores the relevance of hybridization in the origin of new virulent lineages (Mixão and Gabaldón, 2018). The level of genetic variability among *C. inconspicua* hybrid strains is high, including distinct levels of aneuploidies and the presence of mitochondrial heterogeneity. This suggests that *C. inconspicua* hybrids are plastic and prone to adapt to new environments (Mixão and Gabaldón, 2018). Given the medical importance of this species, this should represent a special concern, as this high genomic plasticity may also correlate to a larger phenotypic diversity and a higher propensity to adapt to antifungal drugs and develop new resistances. Thus, more studies to identify new hybrid pathogens, as well as to try to understand how they shape their genomes, and how they can adapt to new environments should be performed. Indeed, it would be very interesting to analyze the genome of environmental strains, to understand if they are also hybrids, or if the hybridization event was the trigger that made this species become a pathogen, as it is suggested for *C. metapsilosis* (Pryszcz et al., 2015).

## Data Availability

Raw sequencing reads generated for this study can be found in SRA under the BioProject Accession No. PRJNA517794. Genome assembly and annotation are available under the same BioProject.

## Author Contributions

AH, TB, and CL-F provided the strains and strain information. AH and CL-F performed susceptibility experiments. ES extracted DNA and prepared material for sequencing. VM performed all bioinformatics analyses. TG supervised the study. TG and VM wrote a first draft of the manuscript. All authors contributed to the final manuscript.

## Conflict of Interest Statement

The authors declare that the research was conducted in the absence of any commercial or financial relationships that could be construed as a potential conflict of interest.

## Footnotes

1 http://www.bioinformatics.babraham.ac.uk/projects/fastqc/

2 http://broadinstitute.github.io/picard/
