# Supplementary material for: Whole-Genome Sequencing of the Opportunistic Yeast Pathogen Candida inconspicua Uncovers Its Hybrid Origin
Source: Front Genet. 2019 Apr 25;10:383. doi: 10.3389/fgene.2019.00383 (PMC6494940; doi:10.3389/fgene.2019.00383)
Supplement: Supplementary file 1 [file Data_Sheet_1.pdf]

Supplementary file 1. List of species used for phylome reconstruction and enrichment analysis results for genes specifically duplicated in *C. inconspicua.*

Table A. List of proteomes used for *C. inconspicua* phylome reconstruction, with species information, proteome version used, number of longest isoforms and number of all isoforms. * - species used as seed

Species	Proteome	Longest isoforms	Isoforms
Candida orthopsilosis	CANO9.1	5,676	5,676
Candida metapsilosis	273372.1	5,960	6,423
Ashbya gossypii	ASHGO.6	4,749	4,749
Kuraishia capsulata	317047.2	6,027	6,027
Clavispora lusitaniae	CLALS.1	5,937	5,937
Lodderomyces elongisporus	LODEL.2	5,782	5,782
Blastobotrys adeninivorans	BLAAD.2	6,118	6,118
Candida tenuis	CANTE.1	5,527	5,527
Pichia kudriavzevii	ISSOR.4	5,088	5,088
Pichia pastoris	PICPA.2	5,072	5,072
Pichia stipitis	PICST.3	5,797	5,797
Saccharomyces cerevisiae	YEAST.9	5,825	5,825
Yarrowia lipolytica	YARLI.5	6,472	6,472
Debaryomyces hansenii	DEBHA.5	6,264	6,264
*Candida inconspicua**	52247.1	5,079	5,079
Candida albicans	CANAL.6	5,139	5,139
Candida glabrata	CANGA.6	5,188	5,188
Candida parapsilosis	CANPA.2	5,784	5,784
Candida tropicalis	CANTR.1	6,230	6,230
Candida dubliniensis	CANDC.1	5,819	5,828
Spathaspora passalidarum	619300.1	5,973	5,973
Ogataea parapolymorpha	OGAPD.1	5,338	5,338

Table B. GO enrichment results for *C. inconspicua* duplicated proteins, with indication of GO term, function, *p*-value considering all *C. inconspicua* proteins and *p*-value when considering only proteins predicted in scaffolds >10kb.

Term category	GO term	Description	***p*-value all proteins**	***p*-value >10kb proteins**
molecular_function	GO:0015171	amino acid transmembrane transporter activity	2.28E-12	2.12E-13
molecular_function	GO:0016491	oxidoreductase activity	2.27E-04	6.02E-05
molecular_function	GO:0050662	coenzyme binding	6.82E-06	4.65E-05
molecular_function	GO:0004523	RNA-DNA hybrid ribonuclease activity	5.13E-04	-
cellular_component	GO:0016021	integral component of membrane	2.78E-08	1.98E-08
biological_process	GO:0046618	drug export	4.88E-07	8.65E-06
biological_process	GO:0055085	transmembrane transport	5.94E-06	7.84E-06
biological_process	GO:0015074	DNA integration	1.36E-10	-
