# Supplementary material for: Whole-Genome Sequencing of the Opportunistic Yeast Pathogen Candida inconspicua Uncovers Its Hybrid Origin
Source: Front Genet. 2019 Apr 25;10:383. doi: 10.3389/fgene.2019.00383 (PMC6494940; doi:10.3389/fgene.2019.00383)
Supplement: Supplementary file 4 [file Data_Sheet_4.pdf]

Supplementary file 4. nQuire histotest results for each strain analyzed in this work.

110_10
Diploid:
Norm SSR: 0.0552134
y-y slope: -0.13725, with std.Err: 0.0382047
r^2: 0.179486

Triploid:
Norm SSR: 0.00637872
y-y slope: 0.442364, with std.Err: 0.0212552
r^2: 0.880116

Tetraploid:
Norm SSR: 0.0206854
y-y slope: -0.556965, with std.Err: 0.0622311
r^2: 0.575849

1282
Diploid:
Norm SSR: 0.0560525
y-y slope: -0.173305, with std.Err: 0.0200732
r^2: 0.558186

Triploid:
Norm SSR: 0.0110048
y-y slope: 0.265766, with std.Err: 0.027109
r^2: 0.619626

Tetraploid:
Norm SSR: 0.0106076
y-y slope: -0.042047, with std.Err: 0.0681987
r^2: 0.00640143

14ANR23920
Diploid:
Norm SSR: 0.00796541
y-y slope: 0.575849, with std.Err: 0.0179947
r^2: 0.945525

Triploid:
Norm SSR: 0.0462348
y-y slope: -0.352075, with std.Err: 0.102429
r^2: 0.166841

Tetraploid:
Norm SSR: 0.0186045
y-y slope: 0.207154, with std.Err: 0.172576
r^2: 0.0238392

9_16
Diploid:
Norm SSR: 0.00469496
y-y slope: 0.689673, with std.Err: 0.0188645
r^2: 0.957724

Triploid:
Norm SSR: 0.0553508
y-y slope: -0.438739, with std.Err: 0.120707
r^2: 0.182955

Tetraploid:
Norm SSR: 0.0240685
y-y slope: 0.231343, with std.Err: 0.205666
r^2: 0.0209952

CBS180
Diploid:
Norm SSR: 0.00405022
y-y slope: 0.69373, with std.Err: 0.0111912
r^2: 0.984878

Triploid:
Norm SSR: 0.0559558
y-y slope: -0.463219, with std.Err: 0.117936
r^2: 0.207277

Tetraploid:
Norm SSR: 0.0240711
y-y slope: 0.210661, with std.Err: 0.204348
r^2: 0.0176938

CI1
Diploid:
Norm SSR: 0.00634326
y-y slope: 0.64761, with std.Err: 0.0241121
r^2: 0.924395

Triploid:
Norm SSR: 0.051284
y-y slope: -0.376603, with std.Err: 0.117842
r^2: 0.147562

Tetraploid:
Norm SSR: 0.0232117
y-y slope: 0.17607, with std.Err: 0.197344
r^2: 0.0133122

CNM_CL6867
Diploid:
Norm SSR: 0.0522364
y-y slope: -0.131567, with std.Err: 0.020434
r^2: 0.412677

Triploid:
Norm SSR: 0.0109143
y-y slope: 0.255593, with std.Err: 0.019971
r^2: 0.735181

Tetraploid:
Norm SSR: 0.0111045
y-y slope: -0.104353, with std.Err: 0.0588597
r^2: 0.0505799

IUM_96-0030
Diploid:
Norm SSR: 0.0499344
y-y slope: -0.10266, with std.Err: 0.0231815
r^2: 0.249477

Triploid:
Norm SSR: 0.00986203
y-y slope: 0.283758, with std.Err: 0.0123337
r^2: 0.899713

Tetraploid:
Norm SSR: 0.013371
y-y slope: -0.248912, with std.Err: 0.0512343
r^2: 0.285742

LL867
Diploid:
Norm SSR: 0.056972
y-y slope: -0.17964, with std.Err: 0.0232311
r^2: 0.503346

Triploid:
Norm SSR: 0.00827729
y-y slope: 0.348859, with std.Err: 0.0154679
r^2: 0.896067

Tetraploid:
Norm SSR: 0.0145952
y-y slope: -0.271285, with std.Err: 0.0658031
r^2: 0.223648

NRZ_BK_345

Diploid:
  Norm SSR: 0.0507581
  y-y slope: -0.117858, with std.Err: 0.0177256
  r^2: 0.428347

Triploid:
  Norm SSR: 0.0109478
  y-y slope: 0.244626, with std.Err: 0.0122517
  r^2: 0.871086

Tetraploid:
  Norm SSR: 0.0124873
  y-y slope: -0.217439, with std.Err: 0.0449416
  r^2: 0.284057

UCSC_1590

Diploid:
  Norm SSR: 0.0584748
  y-y slope: -0.189152, with std.Err: 0.0281812
  r^2: 0.43297

Triploid:
  Norm SSR: 0.00782014
  y-y slope: 0.380578, with std.Err: 0.022632
  r^2: 0.827372

Tetraploid:
  Norm SSR: 0.0168089
  y-y slope: -0.365686, with std.Err: 0.0701593
  r^2: 0.315285
