# Supplementary material for: Whole-Genome Sequencing of the Opportunistic Yeast Pathogen Candida inconspicua Uncovers Its Hybrid Origin
Source: Front Genet. 2019 Apr 25;10:383. doi: 10.3389/fgene.2019.00383 (PMC6494940; doi:10.3389/fgene.2019.00383)
Supplement: Supplementary file 5 [file Data_Sheet_5.pdf]

<u>Supplementary file 5.</u> <u>Recent recombination events detected by fastGEAR.</u>

<u>31 RECENT RECOMBINATION EVENTS</u>

Start	End	DonorLineage	RecipientStrain	log(BF)	StrainName
3293158	3308070	1	1	8.9	110_10
4262783	4283632	3	1	3.3	110_10
793345	797242	3	2	123.4	IUM_96-0030
3792100	3797612	3	2	65.5	IUM_96-0030
3797613	3798580	1	2	76.2	IUM_96-0030
4033845	4037235	1	2	30.7	IUM_96-0030
4800664	4809629	1	2	321.1	IUM_96-0030
5019580	5024822	3	2	434.0	IUM_96-0030
6038588	6040441	1	2	267.7	IUM_96-0030
8495868	8497262	3	2	45.7	IUM_96-0030
143244	144673	2	3	39.5	14ANR23920
192886	208825	3	3	59.1	14ANR23920
1236521	1236853	3	3	91.5	14ANR23920
4028920	4032956	2	3	28.1	14ANR23920
7869680	7875308	2	3	108.7	14ANR23920
1034587	1040329	3	4	15.9	1282
3293158	3308070	1	4	8.5	1282
3789877	3797612	3	4	182.9	1282
3797613	3798580	1	4	75.0	1282
5018802	5027086	3	4	427.6	1282
7486720	7487145	3	4	79.9	1282
793345	797242	3	6	128.2	CBS180
3785659	3797612	3	6	171.6	CBS180
3797613	3798580	1	6	75.7	CBS180
324590	379461	3	7	20.1	CI1
2636366	2638298	2	7	240.6	CI1
4982553	4993632	2	7	110.8	CI1
6036671	6037896	3	7	76.1	CI1
1979365	2002319	3	8	30.7	9_16
7479197	7480860	3	8	12.6	9_16
7480861	7496785	2	8	253.6	9_16
3797613	3798246	1	9	60.5	CNM_CL6867
64253	66739	3	11	22.8	NRZ_BK_345
3365734	3377735	3	11	17.8	NRZ_BK_345
3681675	3726874	3	11	2.7	NRZ_BK_345
5026038	5029477	1	11	35.2	NRZ_BK_345
5864399	5866829	3	11	44.6	NRZ_BK_345
6557895	6571199	3	11	40.4	NRZ_BK_345
7000511	7001533	3	11	42.2	NRZ_BK_345
