# Supplementary material for: Whole-Genome Sequencing of the Opportunistic Yeast Pathogen Candida inconspicua Uncovers Its Hybrid Origin
Source: Front Genet. 2019 Apr 25;10:383. doi: 10.3389/fgene.2019.00383 (PMC6494940; doi:10.3389/fgene.2019.00383)
Supplement: Supplementary file 6 [file Image_1.pdf]

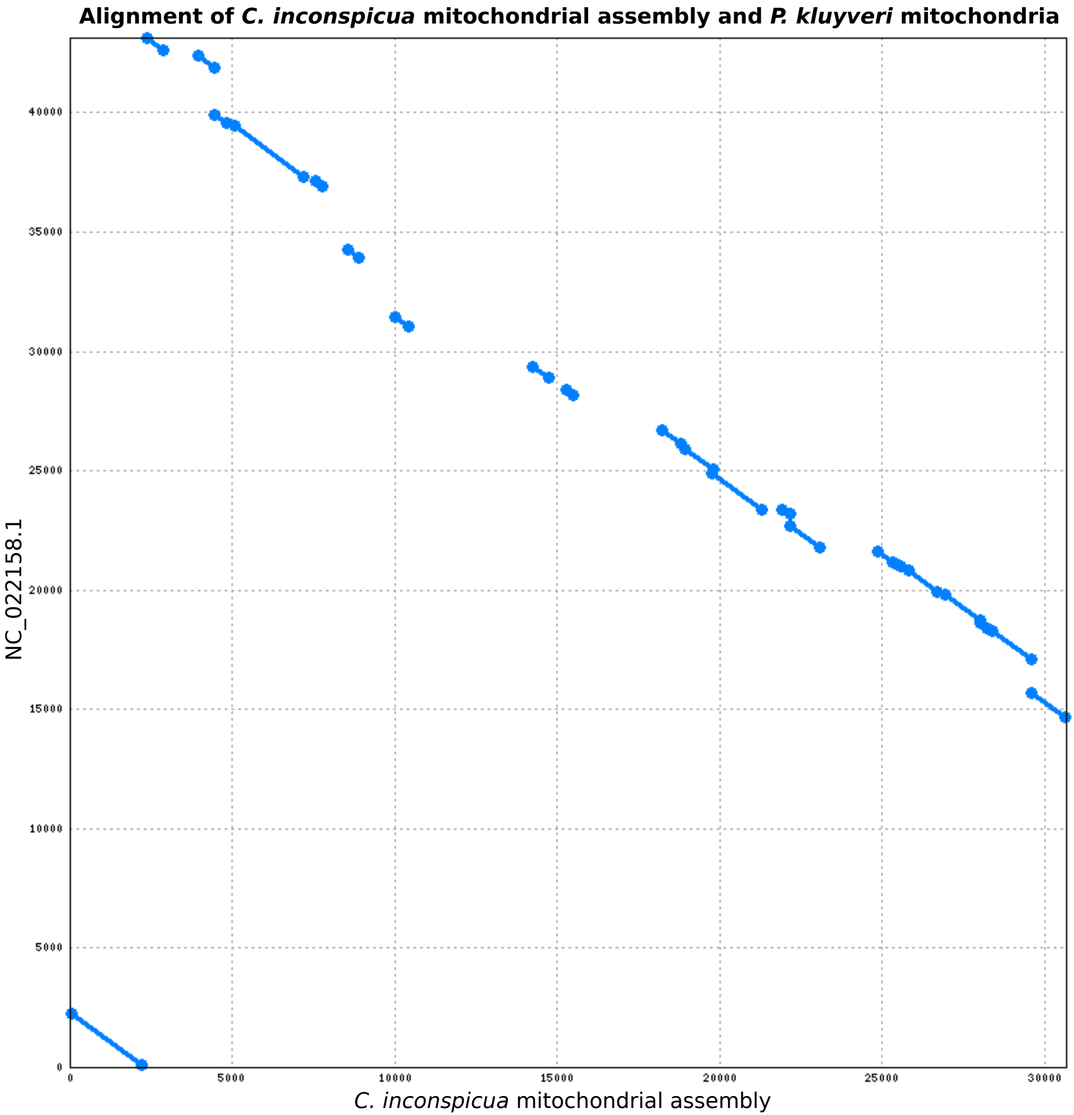

Supplementary Fig1. Alignment of the 31kb region of *C. inconspicua* mitochondrial genome and the complete mitochondrial genome of *P. kluyveri*.
