# Supplementary material for: Whole-Genome Sequencing of the Opportunistic Yeast Pathogen Candida inconspicua Uncovers Its Hybrid Origin
Source: Front Genet. 2019 Apr 25;10:383. doi: 10.3389/fgene.2019.00383 (PMC6494940; doi:10.3389/fgene.2019.00383)
Supplement: Supplementary file 7 [file Image_2.pdf]

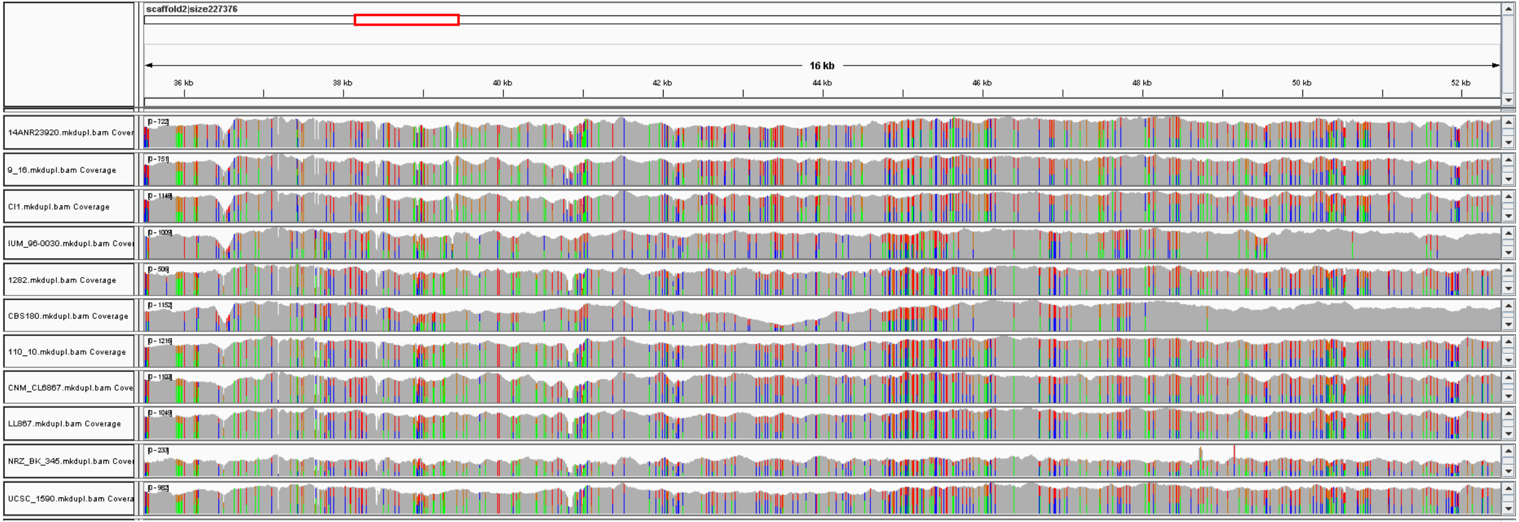

Supplementary Fig2. IGV image of a 16kb region of *C. inconspicua* scaffold 2 for the eleven strains studied in this project. Each row represents the genome coverage track. Colors indicate polymorphic positions.
