# Supplementary material for: Whole-Genome Sequencing of the Opportunistic Yeast Pathogen Candida inconspicua Uncovers Its Hybrid Origin
Source: Front Genet. 2019 Apr 25;10:383. doi: 10.3389/fgene.2019.00383 (PMC6494940; doi:10.3389/fgene.2019.00383)
Supplement: Supplementary file 8 [file Image_3.pdf]

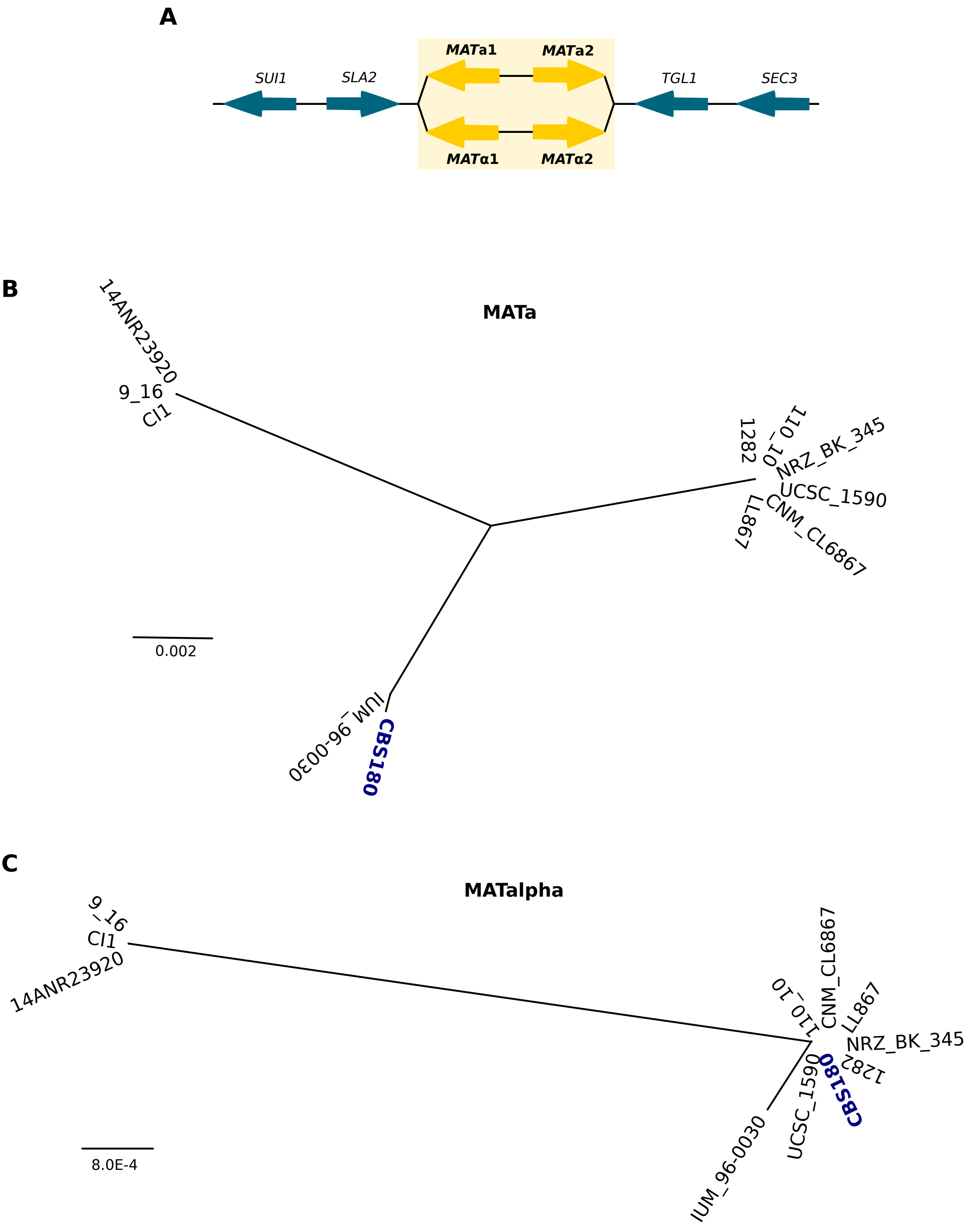

Supplementary Fig3. Analysis of *C. inconspicua* MAT locus. **(A)** Schematic representation of the MAT locus (yellow), with arrows indicating the strand in which the respective gene is coded. **(B)** Phylogeneit tree of MATa. **(C)** Phylogenetic tree of MATalpha.
