# Supplementary material for: Whole-Genome Sequencing of the Opportunistic Yeast Pathogen Candida inconspicua Uncovers Its Hybrid Origin
Source: Front Genet. 2019 Apr 25;10:383. doi: 10.3389/fgene.2019.00383 (PMC6494940; doi:10.3389/fgene.2019.00383)
Supplement: Supplementary file 9 [file Image_4.pdf]

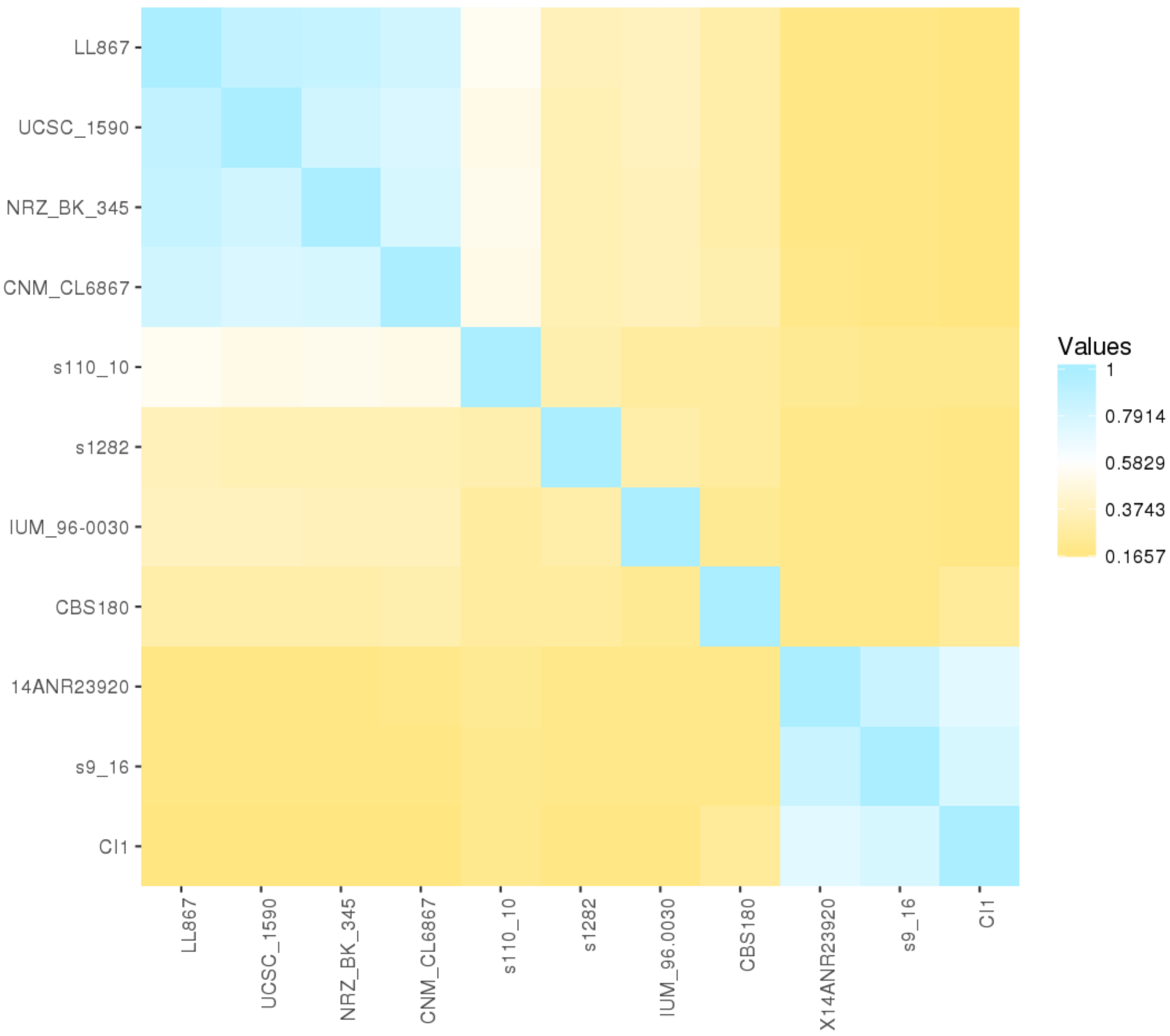

Supplementary Fig4. Heatmap of the jaccard metric obtained for each pairwise comparison between shared LOH positions in *C. inconspicua* strains. The gradient between yellow and blue represents the different jaccard metric values obtained, from the lowest to the highest, respectively.
