# Supplementary material for: Whole-Genome Sequencing of the Opportunistic Yeast Pathogen Candida inconspicua Uncovers Its Hybrid Origin
Source: Front Genet. 2019 Apr 25;10:383. doi: 10.3389/fgene.2019.00383 (PMC6494940; doi:10.3389/fgene.2019.00383)
Supplement: Supplementary file 10 [file Image_5.pdf]

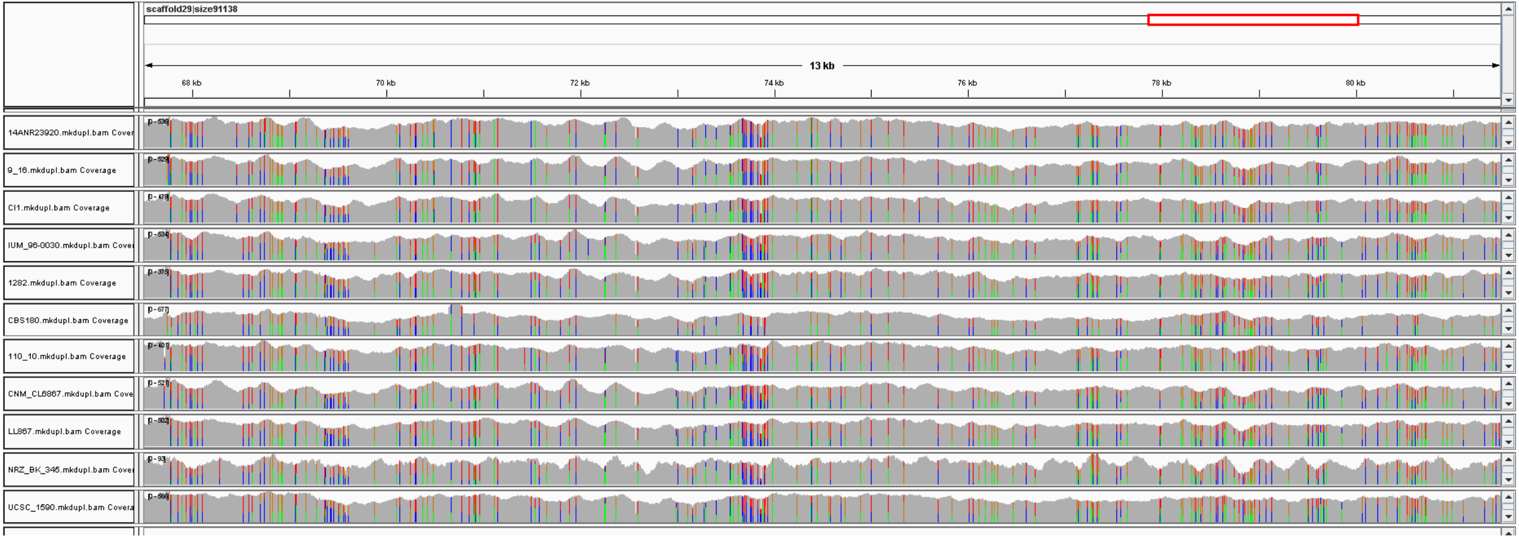

Supplementary Fig5. IGV image of a 13kb region of *C. inconspicua* scaffold 29 for the eleven strains studied in this project. Each row represents the genome coverage. Colors indicate polymorphic positions. In this region, strains from the two clades present similar patterns of LOH and heterozygosity.
