# Supplementary material for: Whole-Genome Sequencing of the Opportunistic Yeast Pathogen Candida inconspicua Uncovers Its Hybrid Origin
Source: Front Genet. 2019 Apr 25;10:383. doi: 10.3389/fgene.2019.00383 (PMC6494940; doi:10.3389/fgene.2019.00383)
Supplement: Supplementary file 11 [file Image_6.pdf]

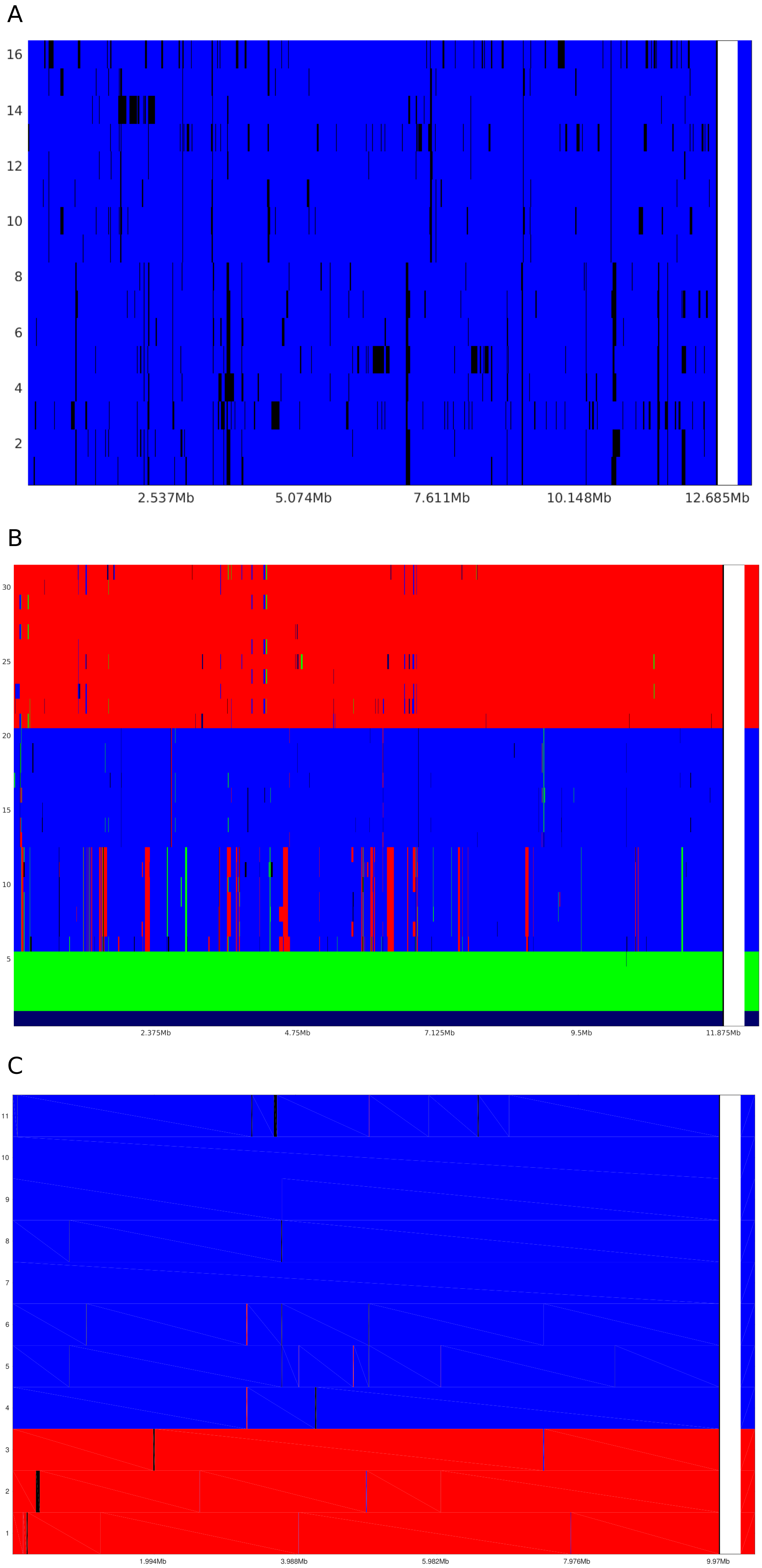

Supplementary Fig6. Recent recombination events predicted by fastGEAR. For each plot, different lineages are represented by different colors, and strains are ordered according to the predicted clusters. **(A)** Prediction for a 12.6Mb alignment of all homozygous positions of 16 *C. metapsilosis* isolates, where only one lineage was identified, consistent with a single hybridization event; **(B)** Prediction for a 11.8Mb alignment of all homozygous positions of 31 *C. orthopsilosis* isolates, where four lineages were identified, consistent with multiple hybridization events; **(C)** Prediction for a 10Mb alignment of all homozygous positions of 11 *C. inconspicua* isolates, where two lineages were identified, one corresponding to the phylogenetic clade 1 (red) and the other to the phylogenetic clade 2 (blue).
