# Supplementary material for: Whole-Genome Sequencing of the Opportunistic Yeast Pathogen Candida inconspicua Uncovers Its Hybrid Origin
Source: Front Genet. 2019 Apr 25;10:383. doi: 10.3389/fgene.2019.00383 (PMC6494940; doi:10.3389/fgene.2019.00383)
Supplement: Supplementary file 12 [file Image_7.pdf]

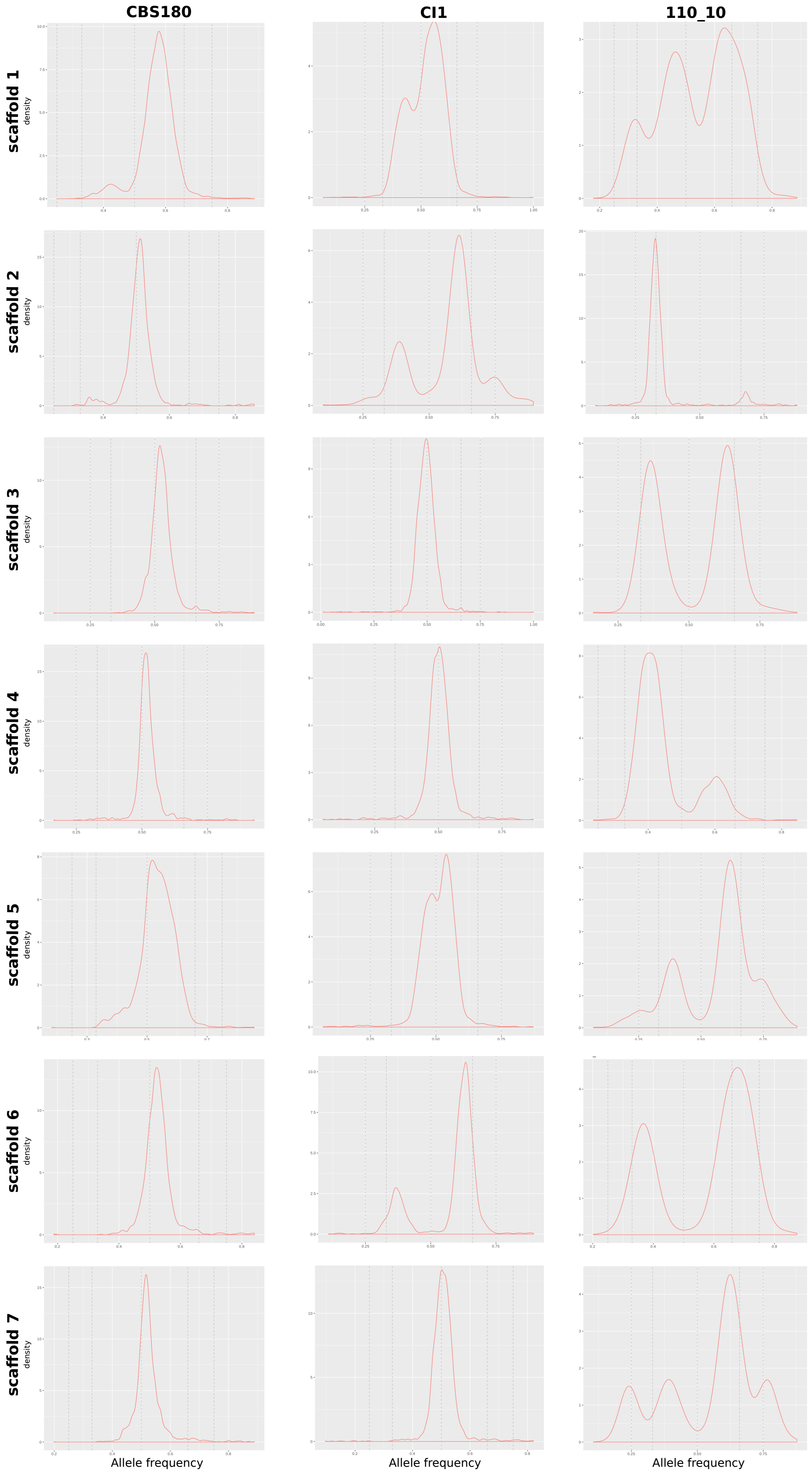

Supplementary Fig7. Density of the allele frequency in seven scaffolds of *C. inconspicua*. Results for two mainly diploid strains (CI1 - clade 1, and CBS180 - clade 2) and for a strain with ambiguous ploidy level (110_10) are shown. Each row represents a different scaffold, and each column a different strain.
