# Supplementary material for: Whole-Genome Sequencing of the Opportunistic Yeast Pathogen Candida inconspicua Uncovers Its Hybrid Origin
Source: Front Genet. 2019 Apr 25;10:383. doi: 10.3389/fgene.2019.00383 (PMC6494940; doi:10.3389/fgene.2019.00383)
Supplement: Supplementary file 13 [file Image_8.pdf]

A

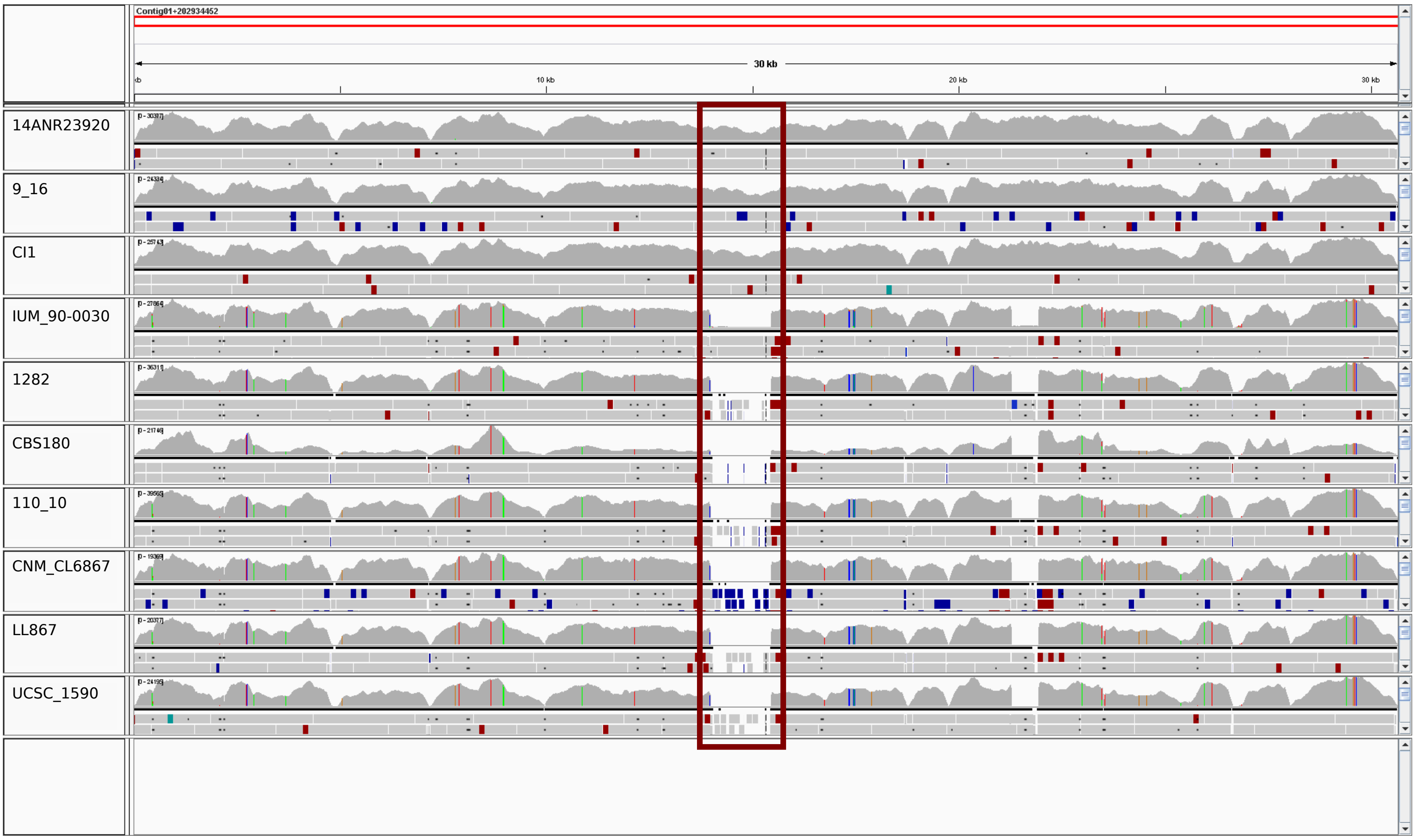

B

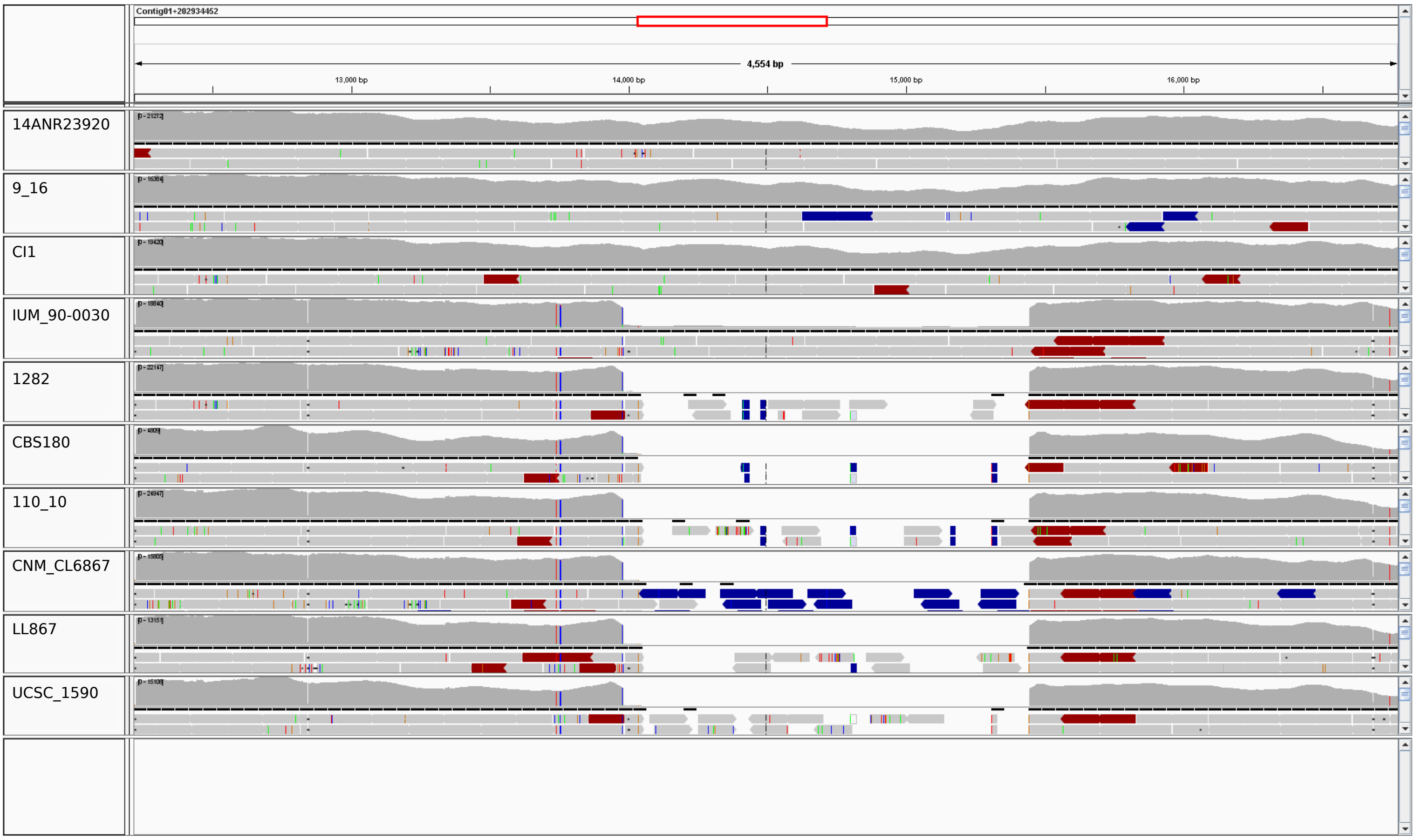

Supplementary Fig8. IGV coverage tracks of the 31kb mitochondrial assembly of C. inconspicua for all strains. Colors indicate polymorphic positions. **(A)** Overview of the entire mitochondrial scaffold, with a major 1.5kb deletion highlighted in red; **(B)** Zoom in on the major deletion highlighted in (A).
