# Supplementary material for: Whole-Genome Sequencing of the Opportunistic Yeast Pathogen Candida inconspicua Uncovers Its Hybrid Origin
Source: Front Genet. 2019 Apr 25;10:383. doi: 10.3389/fgene.2019.00383 (PMC6494940; doi:10.3389/fgene.2019.00383)
Supplement: Supplementary file 14 [file Image_9.pdf]

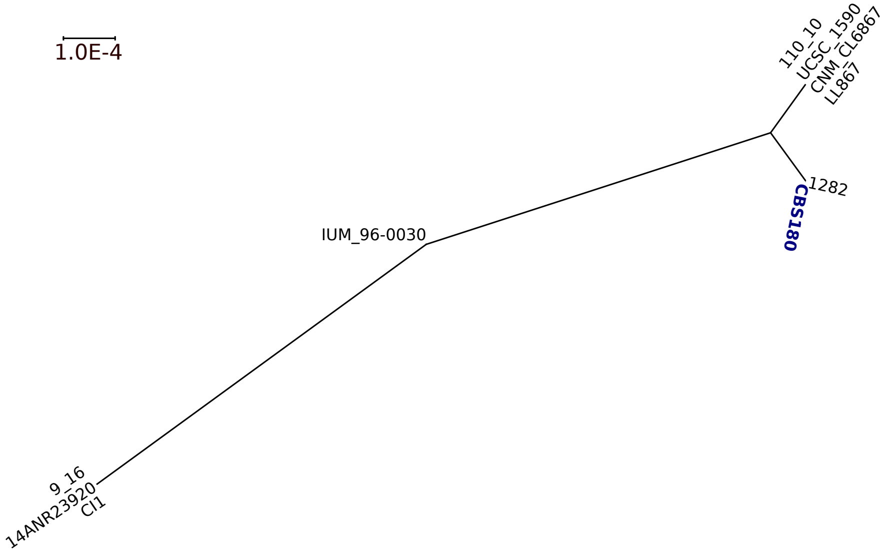

Supplementary Fig9. NJ tree of the alignment of the reconstructed mitochondrial scaffold for each *C. inconspicua* strain. *C. inconspicua* type strain (CBS180) is highlighted in blue.
